# Conditional cash transfers in OECD countries: a realist synthesis

**DOI:** 10.3389/fsoc.2023.1202430

**Published:** 2023-09-14

**Authors:** Francesco Mazzeo Rinaldi, Liliana Leone

**Affiliations:** ^1^Department of Political and Social Sciences, University of Catania, Catania, Italy; ^2^CEVAS, Rome, Italy

**Keywords:** realist synthesis, conditionality, activation, poverty, minimum income

## Abstract

Conditional Cash Transfers (CCTs) schemes have been adopted mostly in low-income countries as a tool to break the vicious cycle of poverty transmission. Although their use is controversial, behavioral conditionalities have also been widely used in welfare-to-work strategies, minimum income scheme, and labor market “activation” policies in OECD countries. The paper presents the results of a Realist Review to synthesize the evidence of CCTs related to work conditionality, delivered in OECD countries. The evaluation literature of 23 selected CCT programs was analyzed by reconstructing Context-Mechanism-Outcome configurations. The main findings show that CCTs can be an effective counterbalance to work disincentives introduced by welfare measures. The unintended negative impacts, the role of sanctioning, and the causal pathways that may affect the most disadvantaged people and their children are discussed.

## 1. Introduction

Since the 90s, the use of welfare conditionalities, which are to be understood as behavioral incentives and usually as work-related requirements, played a crucial role in the policy design of development policies and anti-poverty programs in Latin America and successively rapidly spread across Asia and Africa (Coady et al., 2004; Samson, 2009; Aber and Rawlings, 2011; Evans and Papova, 2014; World Bank, 2015). According to a definition of the World Bank (Fiszbein and Schady, 2009), Conditional Cash Transfer (CCTs) programs aim to reduce current poverty levels through cash transfer to the poor and decrease future poverty through investments in human capital. CCTs programs may be framed as a mixture of negative and positive financial incentives and fiscal measures accompanied by support for employment and social services. Recipients, families, and or individuals are requested to adhere to certain behaviors or achieve the required performance to keep their rights to cash transfers and other benefits such as free access to childcare services or housing benefits.

Literature reviews of CCTs' effects in advanced economies focus on some policy areas, such as education and childcare services (Tárki, 2014; Medgyesi, 2016) and welfare to work policies, particularly in England's welfare reforms (Evans et al., 2003; Watts et al., 2014). As described by the Tárki, Social Research Institute's review in the education sector, several national programs implemented in EU countries adopted CCTs schemes: Kindergarten Allowance (Hungary), Education Maintenance Allowance (UK), the School Allowance student support programme (Belgium), and Child Allowance (Bulgaria). In EU countries significant use is made of CCTs as a tool of work activation within minimum income schemes (Mogstad and Pronzato, 2008; Chemin and Wasmer, 2012). For example, in France a minimum income policy (the Revenu Minimum d'Insertion, RMI), aimed to every individual above age 25 and below a threshold household income, “was initially presented as a mix of welfare and workfare: the transfer would be made conditional on an objective of “insertion” into employment and society, thanks to counseling, provision of incentives and housing allowance” (Mogstad and Pronzato, 2008, p. 5).

Active labor market policies (ALMPs) are a key tool for supporting the re-integration of jobseekers into employment and able to work beneficiaries of minimum income schemes in EU countries are required to participate in active labor market measures and to actively seek employment (European Commission, 2023, p. 70). Minimum income schemes should be designed with strong activation measures for people who are able to work, taking into account policies for temporary care priorities. Moreover, there should be a fair balance overall between incentives and a stronger link with conditions to receive income support and activation measures (EESC., 2022, p. 5–8).

It is important to note that CCTs are not policies *per se* but tools used within different types of programs, such as minimum income and anti-poverty policies, but also health, education, and active labor market policies. While both CCTs and activation policies aim to address social issues and improve wellbeing, their strategies and priorities differ. CCTs programs tackle poverty exclusively through conditional cash transfers. In contrast, activation policies aim to boost employment and self-sufficiency through a broader range of supportive measures, which in some cases may involve financial support. Thus, CCTs typically employ conditionalities, meaning beneficiaries must meet specific requirements to receive cash transfers. Activation policies, however, are centered around actively engaging individuals in employment-related activities, such as job training, job search assistance, or subsidized employment programs. Activation policies vary across countries and regions, and the specific design and implementation of cash transfers as part of these policies can differ. Some countries may prioritize other forms of support, such as subsidized childcare, or job placement services. In contrast, others may rely more on cash transfers as a means of support within their activation policies.

Recent literature suggests that CCTs should be carefully evaluated before national implementation, taking into account cultural contexts and that the interaction with the incentive system of other policies and the underlying mechanisms likely to influence the effectiveness of the programs should be better studied. More research is needed “*on the very effect of conditionality, separate from other program elements”* (Medgyesi and Temesváry, 2013, p. 31) and on the mechanisms by which CCTs influence human behavior (Tárki, 2014, p. 98).

Following the previous claims, this article aims to identify, through a realist review, the mechanisms that, under different circumstances, explain the outcomes of national schemes with CCTs, that is, with economic transfers and subsidies subject to behavioral work-related conditionality aimed at reducing poverty, increasing employment, and investing in human capital. We aimed to explore the consequences of using such tools, even if they were not explicitly referred to by the CCTs acronym in the respective programs, and to shed light on the mechanisms that were triggered in the outcomes produced under different circumstances. The review focuses on programs with CCTs implemented in OECD countries and targeted at families with children.

The operation of CCTs programs can be very diverse and complex. There is a need to understand under what circumstances it would be helpful to include or exclude certain conditionalities, how they interact with countermeasures against social exclusion and unemployment in the most advanced welfare systems, and what kind of enforcement and sanctioning rules might be the most appropriate.

## 2. Materials and methods

### 2.1. Overview of CCTs

CCTs have several objectives, including increasing earned income and employment rates by reducing disincentives to work stemming from previous unconditional welfare subsidies and taxation (see Welfare trap).^1^ CCTs programs differ in many aspects: objectives, type of recipients and targeting methods (means-test, geographical or demographic targeting), types of incentives that can be framed as gains (positive incentives) or losses (negative incentives), conditions related to behavior or performance, size of the transfer, system of monitoring and sanctioning of behavioral conditions (Medgyesi and Temesváry, 2013).

Conditionalities are intended to address behavioral barriers that prevent households from improving their situation and escaping poverty. They should not be confused with requirements for access to the CCTs program. Conditionalities are deemed helpful in:

(a) Inducing people to adopt behaviors (e.g., seeking and maintaining employment) aimed at breaking their dependency on welfare, that they would not otherwise have adopted;
(b) Re-orienting families' choices toward increased investment in human capital making the education of children more affordable/convenient (e.g., reducing school dropout due to financial constraints or in-family cultural differences);
(c) Reducing disincentives to work;
(d) Prevent beneficiaries from wasting money on non-valuable goods.

### 2.2. CCTs and labor market activation approach

Conditionalities are an integral part of the “social activation” strategy, which combines three components or pillars: adequate income support, an inclusive labor market, and access to quality services in an active inclusion strategy (European Commission, 2013a). In the European Union (EU), the labor market “activation” approach has been increasingly adopted, including stricter conditionalities for taking up work, mandatory participation in public work programs, and an increase in sheltered employment (European Commission, 2013b, p. 39). If beneficiaries fail to comply with the requirement (e.g., to be available for work), they are sanctioned with benefit reductions and, in some extreme cases, loss of eligibility for welfare benefits.

Previously unconditional measures have often been criticized for their lack of impact on the employment rates and accused of discouraging job searchers and fostering welfare dependency (Chemin and Wasmer, 2012). Instead, conditionality involves the principle that public support depends on citizens meeting certain behavioral requirements. Those in EU member states are primarily regard labor market “activation” and employability. However, the emphasis on responsibility, reciprocity, and the welfare contract's obligation has been extended to other groups of benefit recipients, becoming increasingly conditional in many states, regardless of the welfare system. Even European countries with a historically more generous welfare system have introduced multiple compliance requirements and sanctions (Frazer and Marlier, 2009). In Denmark, there has been an extension of the working hours required to access ordinary economic subsidies (Anker et al., 2009, p. 8). In Norway, where there used to be a universal minimum income pattern without conditionalities, under the new activation policy, in 2015, “*the government launched proposals for more sanctions for social welfare recipients*” (Westerheim, 2014, p. 10). Nowadays, municipalities have a broader mandate to reduce social assistance benefits if conditions are not met. A relevant critical issue related to the above work activation strategies and the use of conditionality is the increasing phenomenon of in-work poverty in many countries of the European Union. A recent study has shown that active labor market policies with a stronger focus on demanding rather than enabling strategies lead to higher in-work poverty rates (Seikel and Spannagel, 2018). The phenomenon of the working poors refers to low wages, precarious employment and job insecurity (Schraad-Tischler and Schiller, 2016), and the use of welfare-to-work conditionalities may exacerbate it.

### 2.3. The rationale of conditionality and the policy paradigm

A further implicit goal of behavioral conditionality is to make the measure more acceptable from a political perspective. It is widely believed that conditionality appeals strongly to political parties with liberal economic orientation and enhances the acceptability of the measure.

Some scholars (Packwood, 2002; Deeming, 2016) argue that the whole evidence-based approach to policymaking, especially for labor market policies and income-support schemes, has become ideologically driven by the neoliberal paradigm. The CCTs attempts to reconcile minimum income schemes with welfare strategies beyond the “active” neoliberal policy paradigm (Deeming, 2016). The use of conditionalities would support values and beliefs that are compatible with this dominant approach. They have been accused of introducing paternalistic and authoritarian relations between the State and citizens (Schüring, 2010), besides the ethical dilemmas they entail (Watts et al., 2014). However, under certain circumstances, conditionality can be helpful in changing behaviors. At the same time, it also undermines or restricts people's rights (Deeming, 2016, p. 170).

Considerations of political expediency may even guide the choice of methods to be used to evaluate CCTs programs. In the evaluation of Mexico's CCTs Progresa, the questionable emphasis on random control trials (RCT) was a loophole to protect the program's reputation and ensure its survival from political turmoil (Faulkner, 2014). The author critically highlights how socio-political forces may shape quantitative results during and after their production (Faulkner, 2014, p. 231).

### 2.4. The approach

The realist synthesis (Pawson, 2006) was used to review literature, synthesize the results and explore the mechanisms triggered by behavior conditionality across a range of policy domains, from welfare-to-work and “activation” strategies to unemployment benefits, minimum income measures, and school drop-out prevention.

Realist synthesis draws on realist philosophy, which posits that interventions are not universally effective but are contingent upon specific mechanisms and contexts. The approach seeks to uncover the underlying mechanisms that lead to specific outcomes and understand the contextual factors that influence the effects of those mechanisms. The key principle of realist synthesis is “middle-range theory” (Merton, 1968) which aims to identify Context-Mechanism-Outcome configurations (CMOs). CMO configurations describe how specific mechanisms within an intervention interact with particular contexts to produce outcomes. Contexts are not just things, tangible, fixed, observable features or people (material and social) but psychological, organizational, economic, technical, and so on relationships (forces) that interact and influence each other. As contexts and mechanisms interact, they were used to understand how interventions could be targeted at broadly similar contextual conditions or adapted to different contextual conditions (Greenhalgh and Manzano, 2022). Like other systematic reviews, realist synthesis follows a systematic and transparent process of searching, selecting, appraising, and synthesizing evidence from a wide range of sources. The search strategy is designed to capture a broad range of evidence, including not only empirical studies but also theoretical papers, policy documents, and gray literature. The synthesis process involves analyzing and synthesizing the evidence to develop and refine explanatory theories that explain the mechanisms and contexts underlying intervention outcomes (ibid). The approach aims to identify and explain complex causal mechanisms. It explores how contextual factors interact with mechanisms to produce outcomes. However, it does not always provide definitive confirmation of specific causal relationships. The focus is on generating plausible explanations and theories rather than establishing deterministic cause-and-effect relationships.^2^ It also does not prioritize generalizability in the same way as traditional quantitative research. The emphasis is on understanding the underlying mechanisms and identifying patterns of causality within specific contexts rather than making universal claims.

The combination of contexts, underlying mechanisms, and outcomes generates recurrent patterns explaining which implementation processes for recipients succeed in contrasting unemployment, increasing household incomes, or children's wellness and education. The RAMESES guidance (Wong et al., 2013) has been used for reporting the results of the realist synthesis.^3^ The main review questions and sub-questions were:

1. For whom and when do the welfare benefits with work-related conditionality work better, and when might negative impacts be expected? 1.1 how effective are different welfare-to-work approaches?
2. What are the effects of financial sanctioning, and who are the most affected target groups?

We chose the Realist Synthesis approach, as we never intended to identify “best practices for designing and implementing a CCTs.” Best practices “*would imply imposing uniformly to other places, which has been seen to work elsewhere. The real problem in this way of transferring knowledge is the search for the conditions that make (or do not make) it transferable*.” (Stame, 2010, p. 380).

What is at stake here is not whether the same program produces the same effects on different populations. For this, we would have used other literature review methods instead of building, testing, and refining program theories.

A common framework was initially adopted to evaluate public policy, describe program theories underlying the CCTs, and identify their principal mechanisms. We used the classification of policy instruments or government tools through which public sector authorities attempt to trigger social change: the primary instruments are “regulation” (called stick), economic means (called carrot), and information (called sermon)^4^ (Vedung, 1997, p. 123). Different incentive types may converge in a single CCTs program and are classified as positive or negative economic incentives and moral suasion (see Table 1).

**Table 1:** Incentive types.

**Conditionality can be framed as a positive incentive (gain) when it targets specific actions with rewards such as cash transfers, fiscal incentives, or access to other benefits. This is the case when additional financial benefits are conditional on achieving a certain level of school performance or an hourly threshold per week of work. The subject remains free to choose whether or not to take any action. Nevertheless, the hypothesis is that people act based on cost-utility expectations and are prone to behave or achieve a specific performance (e.g., school enrollment and attendance, participation in health prevention)**.
**Conditionality can also be framed as a negative incentive with penalties (loss)** when the instrument includes restrictions and conditions necessary to maintain the benefit with financial sanctions or other monetary loss of benefit as penalties. The welfare recipients are required to fulfill contractual behavioral obligations, and sanctions usually involve the partial or complete loss of social benefits for the short or long term.
**Finally, conditionality can be framed as moral suasion, psychological support, and soft pressure**. This includes attempts to influence people through knowledge dissemination, reasoning, and persuasion with public campaigns to promote counseling and training programs (Vedung, 1997, p. 125). The new emphasis on psychological interventions to “activate” the unemployed may include tasks aimed at changing attitudes, beliefs, and personality. The imposition of “positive affect is routinely imposed in workfare programs via the content of mandatory training courses and through job center or contractor “messaging,” for example, motivational tweets or daily positive emails to claimants” (Friedli and Stearn, 2015, p. 40).

In Table 2, the main policy instruments, namely “economic means and moral suasion,” are associated with expected conditionality mechanisms.

**Table 2:** Classification of policy instruments and basic mechanisms of conditionality.

**Use of policy instruments**	**Conditionality**	**Expected mechanisms for claimants**
Economic means	+ Fiscal or economic incentives (e.g., entitlement to an income tax credit or tax benefit)	**Expectation of a reward** (Positive incentive, Fiscal credit)
	- Economic disincentives: (economic penalties, entitlement exclusion, and/or loss of benefits)	**Fear of benefit loss** (Negative sanctions, disincentive)
Moral suasion	Mandatory coaching, counseling, training courses, and/or social services. Empowerment approaches	**Recall the pledges/commitment; Motivation and positive expectations growth; “Activation.”**

### 2.5. Literature scoping and initial program theory development

**Figure 1:**
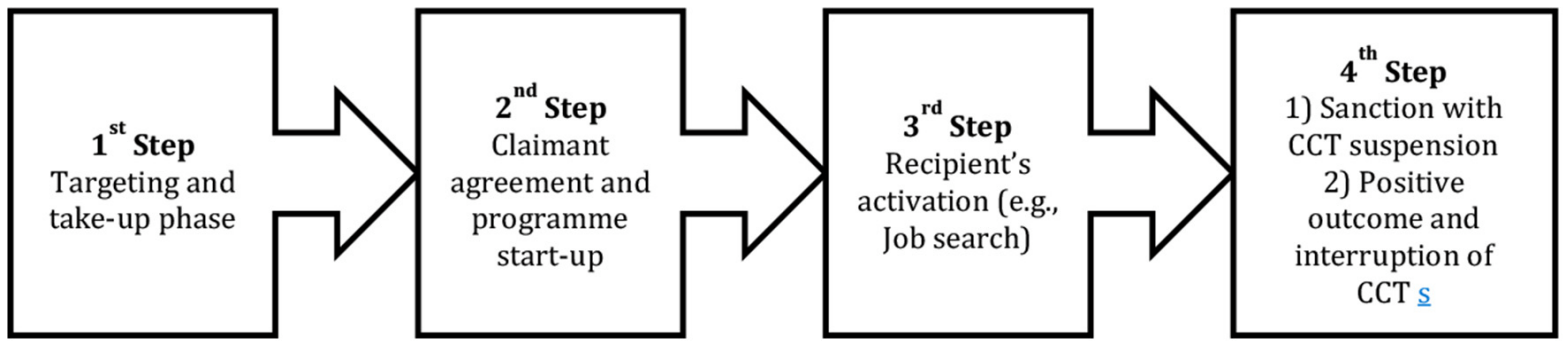
Reconstruction of the initial program theory of a CCTs measure.

The review began with an initial reconstruction of a general CCTs program theory. The initial program theory (Figure 1) was identified during three workshops attended by twenty-five policy-makers and stakeholders (Leone et al., 2017).

The CCTs program theory reconstruction was organized in four main steps: from the targeting and the take-up phase (1st Step) to the entrance of the program with the claimant commitment agreement (2nd step), to intermediate results with complete recipient activation (3rd step), and finally the fourth step with the outcomes or the suspension of the measure (4th step). The stakeholders' interest was focused on the last two steps: (3rd) recipient activation and (4th) suspension of the CCTs measure due to: (1) lack of compliance with the conditionality or (2) the achievement of expected outcomes (e.g., reduction of poverty rate, employment status).

Below are some reviews consulted to sketch the initial program theory of CCTs.

- Hamilton et al. (2001) report the long-term effects of 11 mandatory welfare-to-work programs on welfare recipients and their children. The evaluations came from the United States federally-funded National Evaluation of Welfare-to-Work Strategies (NEWWS), a multi-year study of alternative approaches to help welfare recipients find jobs and advance employment leave public assistance. They cover the period from 1991 to 1999 with a five-year follow-up.
- The review of Huston et al. (2003) illustrates the five-year results of a program implemented in two areas in Milwaukee (Wisconsin, USA), from 1994 through 1998, which aimed to reduce poverty and reform welfare policy (New Hope project for Families and Children).
- Sherman's (2001) review examines child impact findings from 16 local programs in the early and mid-1990s. It comprises 11 programs included in the National Evaluation of Welfare to Work Strategy (NEWWS) and five programs in Minnesota, Wisconsin, Florida, California, and Canada, all evaluated by MDRC through experimental designs.
- The TARKI Social Research Institute 2014 reviewed CCTs implemented in EU Countries and their impacts on children.
- The review of Medgyesi and Temesváry (2013) of CCTs in high-income OECD studied their effects on human capital accumulation.

### 2.6. Process

Primary studies, reviews, and evaluation studies were initially searched through PubMed, EBSCO, and Google Scholar.^5^ According to the realist synthesis approach, source retrieval was a recursive process that began with examining the main systematic reviews of welfare-to-work strategies or minimum income schemes in the OECD countries and identifying evaluation reports and articles about any single program. It continued through the snowball technique with a continuous iterative search. Policy documents were mainly retrieved from European Union, European Commission, World Bank, and National Departments' websites for work and pension (U.K. Department for work and pension and U.S. Department of Labor, France Ministère des Solidarités et des Familles). The search strategy involved intersections of the following terms: Conditional Cash Transfer; CCTs; conditionality; minimum income; welfare to work; activation policies; welfare benefit; outcome; evaluation; enforcement; children wellness; poverty; unemployment.

### 2.7. Selection and appraisal of documents

Three hundred and forty-five electronic references, covering publication years 1999 to 2015, were collected considering all relevant research designs and methods (date last searched: February 2016) and were analyzed. The inclusion of data to inform the program theory development was guided by the RAMESES principles of relevance and rigor (Wong et al., 2013). The following criteria were adopted jointly to select literature and evaluation studies:

- Evaluation studies of CCTs programs with work-related conditionality (such as national schemes of guaranteed minimum income, temporary assistance to needy families, transitional benefits for single mothers, and welfare-to-work programs), aimed at reducing poverty, increasing employment rates and income, and promoting human capital;
- CCTs delivered in OECD countries whose main target groups are families with children.^6^

The quality assurance of the primary source concerns the theory testing process and the appraised inferences rather than the judgment of each contribution. The selection and appraisal of documents were made by two researchers and discussed by the research team. In the last selection step, references were included according to their pertinence to the review's specific sub-questions. Finally, we analyzed the evaluation literature of 21 CCTs programs with two or more evaluation studies for each program.

The typical CMO realist configuration was adopted to compare and examine literature and synthesize evidence. We attempted to identify recurrent patterns of contexts and outcomes, prioritizing the following contexts: (1) mixed approach vs. the work-first approach of welfare-to-work programs; (2) low vs. the high levels of enforcement of work-related conditionality. Successively, we sought to explain these recurrent patterns or demi-regularities through the through an analysis of the mechanisms in which they occurred.

## 3. Results

*Question 1: for whom and when do the welfare benefits with work-related conditionality work better, and when might negative impacts be expected?*

To respond to the first question, we built and analyzed CMO configurations of several welfare programs with work conditionality in the USA, Canada, and the European Union. The results are illustrated in Table 3 and are presented accordingly to the specific sub-question.

**Table 3:** Work conditionality in welfare programs: mechanisms, outcomes, and children's wellness.

**Program and references**	**Context**	**Mechanism**	**Outcome**
(1) TANF– Welfare –to-Work-Strategy USA (Hamilton, 2000, 2002; Sherman, 2001) (2) TANF and Canadian SSP project (Michalopolus and Schwartz, 2001; Grogger and Karoly, 2007)	Job Search First approach Lack of education and cultural services for children and adolescents	**Fear of benefit loss** (Economic negative incentive) **Mothers Overwhelmed**	+ Employment rate increased - Household Income reduction - Negative effects on the wellness of adolescents
	A mix of first activities (education and job) A more generous benefit to compensate for low-paid jobs (e.g., SSP) Personalized approach with counseling and training. Substitute child care services	**Fear of benefit loss** (Economic negative incentive) **Coaching Motivation growth Activation Emotional and social support**	+ Employment rate increased + Within two years, find a better-paid job + Household income increased by at least 20% + Children 0-11 years fewer emotional problems
(3) New Hope - Wisconsin – USA 1994-1998 (Bos et al., 1999; Huston et al., 2003)	A three year program for families with children under the poverty line. Social support strategies through formal and informal networks and children care services offer/supply. Conditionality: to work 30 hours a week	**Fear of benefit loss** (Economic negative incentive) **Activation** (Agency) **Material, emotional and social support** (emotional and social resources)	+ Household incomes increased + Employment rate increased, and stability of employment + Fewer depression symptoms + Fewer discipline problems with children + Children's positive social behaviors and motivation to study + social support networks increased
(4) Transitional benefit reform for single parent – Norway (Mogstad and Pronzato, 2008)	Introduction of work conditionality in single parents benefit system. Two years of economic incentives for full-time work Loss of benefit after the last child aged six years (instead of 10)	**Fear of benefit loss** (Economic negative incentive) **Self-efficacy** (Loss aversion reduction) **Activation Reduction of “economic disincentive”**	+ Employment rate and + Participation in education or training increased
(5) New Deal for Lone Parents (NDLP) United Kingdom - 2001 Work Focused Interviews (Evans et al., 2003)	Program for single parents with children of schooling age. In 2001 work-focused interviews with personal advisers were introduced A mix of tax credits, allowance, and incentives for people with a job lasting one year, plus an incentive for job seekers.	**Fear of benefit loss** (Economic negative incentive) **Coaching Activation Positive fiscal incentive** (tax credit)	+ Employment rate increased with work focused interview
(6) Revenu Minimum d'Insertion (RMI) compared to RSA (Revenu de solidarité Active), France (Chemin and Wasmer, 2012)	Economic benefit without work conditionalities and neither strict sanctions. Generous benefit (Minimum Income)	**Absence of fear for benefit loss Weak activation Fiscal disincentive** (work doesn't pay)	+ Household income increase - Employment rate decrement of 3% if compared with RSA. - Increment of 5 months of unemployment

Five of the six programs significantly increased employment rates and earnings for long-term recipients. The review of Hamilton (2002) summarizes the long-term effects of 11 mandatory programs from the NEWWS, a multi-year United States study of welfare-to-work strategies.

### 3.1. How effective are different welfare-to-work approaches?

Several studies compared the effects of two alternative approaches within the TANF US Programme. The employment-focused or job-search-first approach gave short-term search assistance and encouraged recipients to find employment quickly. The education-focused approach emphasized longer-term skill-building activities (“Human Capital Development” HCD). The job-first approach moved recipients into jobs more rapidly than the second one. However, the more disadvantaged groups that have benefited from programs with a mixture of early activities generally did better than those in job search programs.

Programs with a mix of first activities may have been more effective at increasing earnings because they used more complex methods to determine who would benefit from the job search and who would benefit from primary education. “*In fact, the programs with a mix of first activities used other criteria, such as scores on tests of basic skills and English proficiency*” (Michalopolus and Schwartz, 2001, p. 60).

According to the evaluation, the mechanism that may explain why programs with a mix of first activities result more effective in increasing earnings is the “correspondence with recipient needs.” They effectively determined who would have benefited from job searching vs. basic education (ibid).

### 3.2. Personalized caseload and integrated case management

Responsiveness to recipient needs, resources, and capabilities is crucial to effective CCTs programs with work conditionality (2nd and 3rd lines of Table 3).

The main typology of mentoring mechanisms (Pawson, 2004, p. 7) explains what may happen in the encounter between welfare recipients and social workers. Like mentoring programs, the relationship between staff and recipients offers emotional and cognitive resources and access to material resources (social benefits). Coaching and activation mechanisms have been observed when services adopt personalized caseload and integrated case management methods, particularly for those most distant from the labor market (e.g., SSP project in Canada, New Hope). In programs where there is a flexible offer of personalized counseling and training activities (Context), coaching and emotional support (Mechanisms) may enhance the attitude of self-efficacy, changing motivations, promoting activation processes, and triggering both positive psychological and economic outcomes (Bos et al., 1999; Michalopolus and Schwartz, 2001; Grogger and Karoly, 2007).

The New Hope project participants stated that the staff gave them the information, motivation, and support they needed to achieve their employment goals. Relationships with the program staff “*were equal to or more important than the financial benefits and services that they received (…)”* (Bos et al., 1999, p. 50).

The New Deal for Lone Parents (NDLP) in the United Kingdom is a program that targets single parents with school-age children and aims to bring lone parents back to work. In 2001 mandatory Work Focused Interviews^7^ (WFI) were introduced for some selected groups claiming income support. The aim was to encourage recipients to address barriers to work by accessing various support options. A coaching mechanism and other positive (fiscal inventive) and negative incentives (fear of loss benefit) may explain the program's increased employment rate. NDLP had a significant additional effect on work entry: the cumulative effect of the program over 9 months was to place over 41% of participants into weekly work of more than 16 h, compared to 15% of matched non-participants (Evans et al., 2003, p. 75).

### 3.3. The reduction of economic and fiscal disincentives to work

The mechanism of “economic or fiscal disincentive to work” is crucial to the following welfare benefit reforms. The absence of a negative economic incentive (fear of benefit loss) of the first French national unconditional minimum income scheme, Revenu Minimum d'Insertion (RMI), and the fiscal disincentive due to the reduction of the benefit proportional to work income are considered two critical mechanisms. They might explain the lower employment rate obtained, when compared to the subsequent minimum income scheme named Revenu de solidarité Active (Chemin and Wasmer, 2012). According to the evaluators, the Transitional Benefit, the unconditional generous out-of-work welfare scheme tailored to lone mothers in Norway, acted as a disincentive to enter the labor market. In Norway's welfare reform with the Transitional Benefit (1998), several new conditions were introduced for welfare eligibility (upper age limit of the youngest child, time limits on participation) to contrast the low work incentive in relation to the benefit. The benefit was also linked to activity requirements, including employment and education enforced by non-compliance sanctions. Moreover, the maximum benefit level (€855 after the reform) was also increased (Mogstad and Pronzato, 2008, p. 3). The Norwegian reform increased earnings and education reduced the poverty rate, and lowered welfare caseloads and, therefore, the government's financial burden (ibid, p. 25).

*Question 1.2 Do conditionalities about work increase household income and, thus, the wellbeing of the children?*

Adult employment alone – a more traditional yardstick for judging welfare-to-work programs – shows little connection with child wellbeing. There is evidence that in contexts where there is a lack of educational services for children and a low level of parental education, the “Job search first approach” adopted in the welfare-to-work strategy (TANF Programme) may produce negative effects on teenagers' wellness, together with a reduction of the household income. Single parents, usually mothers, reported being overwhelmed by full-time, poorly paid jobs and needing to care for their younger children, sometimes losing control over their older children. It should be noted that teenagers (primarily males) are at higher risk. Usually, for young people aged 14–18 years, there is a lack of good quality non-formal education services and a risk of being “Not in Education, Employment or Training” (NEET).

Two reviews (Hamilton, 2000; Sherman, 2001) examine child impact findings from sixteen local welfare-to-work TANF programs confirming the pivotal role of income. Across different sites, impacts on household income “*spanned a wide range, from a 20 percent increase in Portland to a 15 percent decrease on one of the two Grand Rapids programs. (…) Impacts on children also varied dramatically”* (Sherman, 2001, p. 7).

The results, synthesized in Table 4, suggest that the most successful welfare programs for children have improved parents' income by rewarding and encouraging them to work. All the welfare-to-work programs that lifted participants' average incomes by 5% or more had “mostly positive” effects on children. Furthermore, every program that reduced income by 5% or more had negative behavioral and emotional negative effects on children.

**Table 4:** Programs categorized by approach, household income, effects on children, and enforcement level.

**Program**	**Household income after the program**	**Effects on children**	**Effects on children School performance**	**Effects on children Health and Safety**	**Total effects**	**Enforcement**
1. Portland, OR (NEWWS)	Increase by more than 5%	+			++	Medium/high
2. Minnesota Family Investment Program (MFIP)	Increase by more than 5%	+	+		+++	n.a.
3. New Hope	Increase by more than 5%	+	+		+++	n.a.
4. Canada's Self-Sufficiency Project (SSP)	Increase by more than 5%	- (for adolescents)	+ (- for adolescents)	+	++	n.a.
5. Atlanta, GA (1) (NEWWS) LFA - Job search first	Increase by more than 5%	+			++	High
6. Atlanta, GA (2) (NEWWS) HCD	No effect or under 5%				No effects	High
7. Oklahoma City, OK (NEWWS)	No effect or under 5%	-			-	Low
8. Los Angeles Jobs-First GAIN	No effect or under 5%	+			+	n.a
9. Columbus, OH (A) (NEWWS) Traditional case management	No effect or under 5%			-	-	High
10. Detroit, MI (NEWWS)	No effect or under 5%				No effects	Low
11. Columbus, OH (B) (NEWWS) Integrated case management	No effect or under 5%	+	+		++	High
12. Florida Family Transition Program (FTP)	No effect or under 5%	+		+	++	n.a.
13. Riverside, CA (2) (NEWWS) HCD	Lowered over than 5%	-			-	High
14. Riverside, CA (1) (NEWWS) LFA - Job search first	Lowered over than 5%	- -	+		- - +	High
15. Grand Rapids, MI (2) (NEWWS) HCD	Lowered over than 5%	-		-	- -	Very High
16. Grand Rapids, MI (1) (NEWWS) LFA - Job search first	Lowered over than 5%	-			-	Very High

n.a. Not analyzed; + = positive outcomes; - = negative outcomes. LFA - Labor Force Attachment is focused on employment, HCD - Human Capital Development is focused on education [Modified from Hamilton et al. (2001); Sherman, 2001, p. 26].

The results are sensitive to children's ages: no programs helped the oldest group (11-to-18 years old), even those that increased income.

*Question 2. What are the effects of financial sanctioning and enforcement of conditionalities, and who are the most affected target groups?*

The findings generally support the notion that a moderate level of enforcement by program officials in welfare-to-work programs is crucial in attaining employment outcomes and motivating individuals who may not voluntarily enter the labor market. The total effects of sixteen programs entered in the NEWWS, illustrated in Table 4 above, are partially related to the degree of conditionality enforcement (e.g., long-term penalties, amount, and frequency of sanctions). We collected data about the enforcement degree in 11 programs: in two, there was a “Very high” enforcement (Grand rapids 2 and 1). In both programs, negative impacts were registered for children. Better effects for children's wellbeing were noted in programs with high conditionality enforcement (but without high sanctioning) (Columbus OH, Atlanta GA, Portland).

As illustrated in the previous paragraph (Table 3 Programmes 3 and 5), moderate pressure to comply with conditionality may have a positive effect because it reinforces and sustains recipients motivations and may favor the empowerment of recipients (Mechanism), offering valuable emotional and material support and access to information.

The initial program theory assumes that the fear of financial sanctions operating within CCTs will promote desired behavior and favorable outcomes. By contrast, when authors distinguished whether the effect is created by the threat of a sanction before the claim (with an impact on take-up rates) or during the claim (fear of benefit loss mechanism), the evidence demonstrated the negative effects of sanctions.

As illustrated in Table 5 in the TANF programs (Grand Rapids 1–2 and Riverside 1–2), robust conditionality enforcement with heavy sanctions and a significant loss of financial subsidies for a long period are associated with adverse outcomes in terms of income, job quality, and unemployment stability (Griggs and Evans, 2010).

**Table 5:** The role of sanctions in CCTs: CMO configuration.

**Program**	**Context**	**Mechanism**	**Outcome**
TANF Temporary Assistance to Needy Families -USA (Hamilton and Scrivener, 1999; Pavetti et al., 2003; GAO, 2010; Griggs and Evans, 2010)	Low quality (at different levels) of orientation and job services	**Lack of understanding of CCTs rules**	High sanction rate
	Disadvantaged target groups with low education and work skills, living in areas less served by public transport	**Economic disincentive**	High sanction rate Excluded by the program after an initial take-up and inclusion
Unemployment Benefit -Switzerland (Arni et al., 2009)	Benefit scheme for unemployed with referral procedure or sanctions	**Lower bargaining power due to sanction** (financial restriction) or **Fear of benefit loss** **Lower expectations**	Individuals accept less favorable job proposals because they need a job within a few weeks. Lower future incomes (−8.6%) Long-term impact (2–5 years) = Higher Employment but lower Income (−1.8%)-and job quality.

Sanctions to be effective should be imposed on individuals with higher education, ready-to-work, past working experiences, more skills, and more chances to enter the labor market. Evidence demonstrated that families with multiple complex problems and younger recipients under 25 years were more likely to have been negatively affected by financial sanctions (Pavetti et al., 2003, p. 17).

A critical factor determining how conditionality influences people's behavior is the degree of knowledge of the rules. About one out of four sanctioned recipients did not clearly understand the conditions because service providers either did not inform them of the rules' complexity or did not understand this (ibid). Most sanctioned families tended to be less able to respect the rules and had more disadvantages (Griggs and Evans, 2010).

In summary, where there is moderate enforcement of conditionality managed by the personal adviser or a job counselor, specific mechanisms (enhancing commitment and recipients' self-efficacy) might strengthen job-search capability leading to a positive outcome (employment and income). Inversely, high sanctions rates negatively affect income and employment rates substantially because (a) they discourage job search and (b) they tend to be addressed to the target group with the most difficulties (context). Moreover, high sanction rates and strict conditions decrease the take-up rate of potential beneficiaries.

An evaluation of the long-term effects of the sanctions in a benefit scheme for the unemployed was carried out in Switzerland (Arni et al., 2009). The study analyzed the data extracted from a public register (unemployment insurance register database UIR) containing information about all individuals registered with public employment services for 1998–2003. Almost 20% of these people had received a warning that, in fewer than half of cases (8.4%) turned into a sanction reduction or suspension of benefits. The penalty affected the total benefits for up to 60 working days. Recipients were pressured by financial constraints and therefore became inclined to lower their expectations (Mechanism) and quickly accept less convenient job proposals. After the first few years of applying the scheme, sanctions were negatively associated with the employment rates and the income average (Outcome) (Arni et al., 2009).

## 4. Discussion

The realist review, whose results have been discussed in this paper, aimed at responding to the following main evaluation questions: (1) For whom and when do the work-related conditionalities work better, and when might negative impacts be expected? (2) What are the effects of financial sanctioning and strict enforcement of conditionalities, and who are the most affected target groups?

The initial rough program theory of CCTs (Figure 1) has been deeply modified and articulated. A more comprehensive number of mechanisms operating in welfare benefits with work-related conditionality have been identified through the realist synthesis approach. The CMO configurations (Table 3) illustrate recurrent contextual factors and circumstances that explain positive and negative outcomes.

The notion of “context” refers to the institutional and policy environment, the specific system of welfare, and characteristics included in the design of the measure (e.g., generosity of the allowance, requirements for inclusion in the benefit, flexible offer of personalized counseling and training activities).

A chain of mechanisms is responsible for the observed outcomes, not limited to the initial five mechanisms (mostly economic) that were assumed (Table 2).

### 4.1. For whom and when the work-related conditionalities work better

The fear of a loss of benefit, that is, the negative economic incentive, cannot alone trigger behavioral changes that lead to positive outcomes for recipients and their children. The mechanism of “activation” of recipients is triggered if they perceive: (a) a positive incentive to work (e.g., the use of tax credit measures) and the elimination of economic disincentives connected to other welfare benefits (e.g., French and Norwegian reforms), (b) flexible solutions with clear rules, (c) a mix of counseling and training services that lead to a growth of self-efficacy and (d) social support for their children.

Practices based on a personalized caseload that identify the barriers to entering the labor market and develop individual action plans are more effective for earnings increase, as they consider the specific needs of definite jobseekers/ householders. Moreover, through the use of mutual commitment of the parties, State, and recipient households, social services and Jobcentres succeed in engaging job seekers.

The progressive decrease of the benefits and an adequate period to test the exit from welfare dependency is crucial to modify attitudes and decisional strategies of the recipients (particularly lone parents), leading to “loss aversion reduction” and investments in the long-term objectives.

### 4.2. Unexpected and expected impacts of CCTs programs on children's wellness

A critical issue in evaluating welfare reforms and CCTs measures is the perspective used by the evaluators to judge the impacts. The review demonstrated that the employment rate of beneficiaries (often used as the main impact indicator for the success of a program) might have a variable connection with child wellbeing and, therefore, with the ultimate goal of interrupting the vicious circle of poverty. Other mechanisms are in operation that may affect the children's wellness, especially that of teenagers who are the most exposed to drop-out phenomena and high risk of NEET (Not in Education, Employment, or Training).

Conditionalities regarding parents' jobs (e.g., search for a job, number of weekly hours) succeed in increasing household income and their children's wellbeing only in those contexts where a mix of favorable factors was active. There are more generous benefits to compensate low paid jobs (e.g., SSP). The services adopt a flexible, personalized approach with counseling and training and avoid pushing recipients toward Job Search First (Portland and Atlanta). Substitutive low-cost public child care services or formal and informal support child care services were offered.

### 4.3. Enforcement of conditionality

The second part of the paper addresses the second question on the effects of conditionality enforcement and sanctions. The enforcement of behavioral conditionalities that encompasses soft or strict monitoring of pledges, and the duration and severity of sanctions applied to recipients, influenced the outcomes and even the take-up rate of the measures. The monitoring system to enforce conditionalities is a critical component of CCTs. Medium enforcement of job conditionalities with low sanctions rates and a mix of fiscal credit and sound quality are associated with activation mechanisms for the most disadvantaged recipients and positive outcomes on employment, income, and children's wellness.

Strict monitoring of conditionalities does not automatically entail high sanctioning rates: on the contrary, strong evidence emerges of the risks of sanctioning the most disadvantaged target groups. Evaluation studies of the Temporary Assistance for Needy Families (TANF) program in the United States demonstrated that a stricter sanction policy led to a take-up reduction with a loss of potential recipients (Pavetti et al., 2003, p. 19). Benefit sanctions have disproportionate effects on vulnerable people and younger age groups (Hamilton and Scrivener, 1999; Pavetti et al., 2003; GAO, 2010; Griggs and Evans, 2010; Dwyer and Wright, 2014). This is because there is a lack of understanding about complex rules and target groups with more practical impediments to accomplishing some requests (e.g., transport difficulties), such as attending a job interview and work-focused counseling.

Job seekers with conditional unemployment benefits are influenced by the constraints and duration of the benefit. The fear of severe restrictions due to referral procedures or benefit interruption may lower the bargaining power of unemployed individuals with long-term negative impacts on the income side. Recipients that are too threatened by the risk of financial restriction are pushed to accept unfavorable job proposals and have adverse long-term outcomes in household income (Arni et al., 2009).

### 4.4. Rights and obligations in the use of conditionality

The key critique of the notion of “responsibilization” - mentioned in the introduction - is a lack of public debate about the rights that should accompany citizenship obligations, mainly for the poorest and least powerful in a society overwhelmed by the increasing conditionality (Lister, 2011). The unemployed are requested to perform specific activation duties, but it doesn't seem that the same compulsion applies in the case of the State's provision of adequate employment services (Bazzani and Singer, 2016).

The “activation” mantra that matches the EU labor policies risks remaining a rhetorical formula if not accompanied by a necessary co-responsibility of all those involved in this area and by providing high-quality public services.

Moreover, the choice of appropriate behavioral conditionalities, in terms of contents, time of application, intensity, and progression of sanctions need to consider the high expenditure for administrative costs (e.g., set-up costs, targeting and enrolment of beneficiaries, operational, monitoring, and evaluation costs) and the cost-efficacy balance for local-level public administrations (Mogstad and Pronzato, 2008; Faber and Koning, 2012). According to White et al. (2013), a potential trade-off exists between administrative costs and the impacts of welfare measures.

Realist synthesis is an evidence-synthesis approach that aims to understand how and why interventions work in specific contexts. We adopted it because its strengths lie in providing a contextual understanding of interventions and identifying underlying mechanisms. This focus on mechanisms gave us insights into the causal processes involved, helping us explain why interventions work or do not work in specific contexts. Realist synthesis offers actionable insights for policy and practice by developing and refining theories. However, limited data availability, especially for emerging or complex interventions, and methodological complexities, such as developing initial program theories, identifying relevant evidence, and synthesizing diverse data sources, can impact the synthesis's comprehensiveness and validity. It is also time-consuming. We had to extrapolate mechanisms from an extensive analysis of different programs' evaluations carried out using qualitative and quantitative methods, often “organized” in different ways than the original evaluation reports. This required continuous discussion within the research group, mainly to minimize interpretive disparity. Thus, interpretation subjectivity and time/resource intensiveness might pose severe limitations.

## 5. Conclusions

Finally, two elements should be mentioned that may open new perspectives for research on the use of behavioral conditionality in welfare measures. The first is the economic context. In many countries experiencing an economic “downturn” and a systematic reduction in job opportunities, specific evaluations should look closely at the potential ineffectiveness or perverse effects of work conditionality. As the review indicates, CCTs with high sanction rates may affect the most disadvantaged target groups: claimants with human capital deficits or facing practical barriers to work are more likely to be sanctioned (Griggs and Evans, 2010). Moreover, discrepancies are emerging between out-of-work conditionality (unemployed claimants) linked to minimum income schemes and real opportunities provided by active labor market policies (e.g., employment and training opportunities, work experience placements) (Lister, 2011; Bazzani and Singer, 2016). The rhetoric of activation policies, which focuses on individual responsibility, emphasizes only one side of the mismatch between labor supply and demand. However, in the absence of an effective active labor policy operating on the other side of the labor supply, there is a lack of opportunities to participate in work-oriented activities to improve employability and the “activation of recipients.”

Secondly, as suggested by other authors (Griggs and Evans, 2010, p. 8), it would be necessary to look more closely at the (potential) positive or negative spillover effects of conditionality on the gray and black economies and on everyday work. The choice to enter the welfare system might create unfavorable conditions for the emergence of “gray economy” even if a means test of family sources of income and assets is applied.

In conclusion, the main results of the review suggest that: (a) the debate on work-related conditionality has not sufficiently focused on effects on children, although CCTs directly affects their wellness and the intergenerational transmission of poverty; (b) empowerment approaches to manage the relationship between the personal adviser and the CCTs recipient, work best for more disadvantaged people; (c) the right to effective labor market services should accompany welfare recipients' obligations, particularly those of the poorest.

## Author contributions

All authors listed have made a substantial, direct, and intellectual contribution to the work and approved it for publication.

## Footnotes

^1^When cost opportunity due to work is too high compared with the financial return, it may arise a perverse incentive not to work may arise.

^2^This involves generative causation, a perspective to understanding causality that focuses on the mechanisms and processes that generate causal effects. Instead of viewing causation solely as regularity or correlation between events, generative causation seeks to uncover the underlying mechanisms or processes that produce or give rise to specific outcomes. Various authors and scholars have discussed and developed generative causation over the years (e.g., Salmon, Shultz, Coleman and Elster).

^3^The RAMESES (Realist and Meta-narrative Evidence Synthesis: Evolving Standards) guidance is a set of methodological guidelines for conducting realist reviews and meta-narrative reviews in social science research. The guidance was developed to address the methodological challenges specific to realist and meta-narrative reviews, helping researchers produce high-quality and reliable evidence syntheses in the social sciences.

^4^Sermon might also be defined by activities that could also share the character of “carrots” (e.g, participation to training programs, if them allow people to improve their position on the labor market).

^5^Although there are other relevant tools for academic research, these three search engines and databases were considered the best combination given the resources available and the specific research needs.

^6^CCTs measures in the education domain (without work-related conditionality) directly addressed to children that aim to promote human capital and health have been excluded (Tárki, 2014; Westhorp et al., 2014).

^7^WFI is a mandatory interview for engaging with customers, which takes place at regular intervals, between lone parents and their Jobcentre Plus adviser (case worker).
